# A Chimeric DNA/RNA Antiparallel Quadruplex with Improved Stability

**DOI:** 10.1002/open.202100276

**Published:** 2022-02-01

**Authors:** Elaina P. Boyle, Levan Lomidze, Karin Musier‐Forsyth, Besik Kankia

**Affiliations:** ^1^ Department of Chemistry and Biochemistry The Ohio State University Columbus OH 43210 USA; ^2^ Center for RNA Biology The Ohio State University Columbus OH 43210 USA; ^3^ Institute of Biophysics Ilia State University Tbilisi 0162 Republic of Georgia

**Keywords:** DNA, quadruplex, thrombin-binding aptamer, stability, RNA

## Abstract

Nucleic acid quadruplexes are proposed to play a role in the regulation of gene expression, are often present in aptamers selected for specific binding functions and have potential applications in medicine and biotechnology. Therefore, understanding their structure and thermodynamic properties and designing highly stable quadruplexes is desirable for a variety of applications. Here, we evaluate DNA→RNA substitutions in the context of a monomolecular, antiparallel quadruplex, the thrombin‐binding aptamer (TBA, GGTTGGTGTGGTTGG) in the presence of either K^+^ or Sr^2+^. TBA predominantly folds into a chair‐type configuration containing two G‐tetrads, with G residues in both *syn* and *anti* conformation. All chimeras with DNA→RNA substitutions (G→g) at G residues requiring the *syn* conformation demonstrated strong destabilization. In contrast, G→g substitutions at Gs with *anti* conformation increased stability without affecting the monomolecular chair‐type topology. None of the DNA→RNA substitutions in loop positions affected the quadruplex topology; however, these substitutions varied widely in their stabilizing or destabilizing effects in an unpredictable manner. This analysis allowed us to design a chimeric DNA/RNA TBA construct that demonstrated substantially improved stability relative to the all‐DNA construct. These results have implications for a variety of quadruplex‐based applications including for the design of dynamic nanomachines.

## Graphical abstract

**DNA→RNA substitutions** in the context of a monomolecular, antiparallel quadruplex, the thrombin binding aptamer (TBA) have been investigated in the presence of either K^+^ or Sr^2+^. Analysis of the stabilizing and destabilizing influences allowed to design a chimeric DNA/RNA TBA construct that demonstrated substantially improved stability relative to the all‐DNA construct.

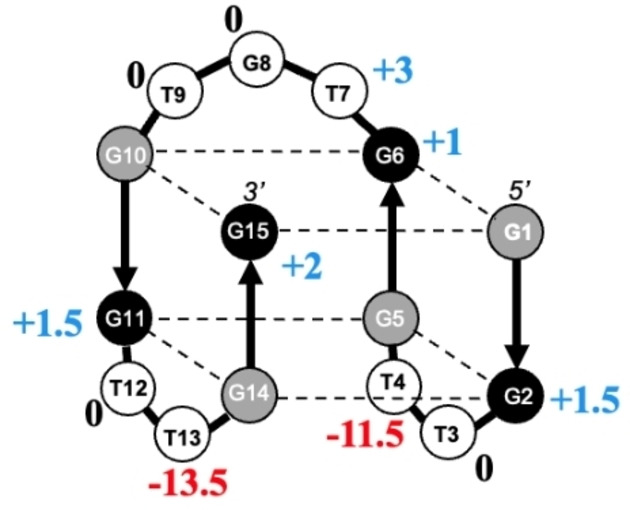

## Introduction

Nucleic acid quadruplexes play critical roles in biology, including their role in the regulation of gene expression.^[1]^ These structures are also widespread in functional nucleic acids such as aptamers^[2]^ and have potential therapeutic and biotechnological applications.^[3]^ The primary structural element within quadruplexes is the guanine (G)‐quartet. The G‐quartet describes a very specific, square planar association of guanines; these structures are stabilized by coordination to a centrally located cation, such as Na^+^ or K^+^. Each G directly interacts with two adjacent Gs using both its Watson‐Crick and Hoogsteen hydrogen bonding faces. In addition, each G has indirect (through the central cation) contact with the diagonally positioned G. Despite these strictly defined recognition/association rules, G‐tetrads can adjust to a myriad of strand‐alignments (i. e., parallel or various antiparallel topologies), which affects groove dimensions and overall size of the quadruplexes. Regardless of strand alignment, the quadruplex always retains its internal G‐tetrad geometry. As a result, DNA quadruplexes are highly polymorphic and the topology of most monomolecular quadruplexes is largely dependent on experimental conditions (i. e., counterion type, pH, water activity), or even conditions present during sample preparation.^[3b]^ Some G‐rich sequences demonstrate simultaneous polymorphism by forming an ensemble of structures with similar free energies but different topologies.^[4]^ The presence of multiple species in solution complicates experimental studies including structural analyses. Sequence modifications may be used to selectively favor a particular quadruplex structure.^[5]^ An improved understanding of thermal stability within quadruplexes, in combination with structural polymorphism, would be beneficial, as structures with enhanced thermodynamic stability may have more predictable behavior.

The 15‐nucleotide (nt) DNA sequence, GGGTGGGTGGGTGGG (G3T), derived from an HIV integrase aptamer,^[6]^ exhibits unusually high stability and structural monomorphism.^[7]^ In the presence of 0.1 mm KCl, it demonstrates cooperative and fully reversible melting curves with a melting temperature, *T*_m_, of 55 °C. G3T folds into a parallel quadruplex comprised of three G‐tetrads with all Gs in the *anti* conformation. The top and bottom G‐tetrads are connected by propeller (or double chain‐reversal) T‐loops.^[8]^ The all‐parallel topology is not affected by the size of cation.[^7^, ^9^] It also tolerates all nt substitutions in loop positions.^[10]^ However, almost any sequence modification within the G‐tetrads, as well as lengthening of the loop sequences, induces a rearrangement of the parallel structure into antiparallel topologies.

Since antiparallel topologies require certain G residues to adopt the thermodynamically less favorable *syn* conformation, they generally possess considerably lower thermal stability than parallel structures.^[8]^ Notably, the RNA sequence ggguggguggguggg (g3u) establishes an identical tertiary structure as its DNA counterpart and is thermodynamically even more favorable with a *T*_m_ of 68 °C (Δ*T*_m_=13 °C).^[11]^ Our previous investigation of the all‐parallel G3T monomolecular quadruplex led to an improved understanding of how specific ribonucleotide substitutions lead to stabilization. However, the majority of monomolecular quadruplexes fold into an antiparallel topology. Therefore, it is important to understand principles of thermodynamic stabilization in the alternate quadruplex fold.

**Figure 1:**
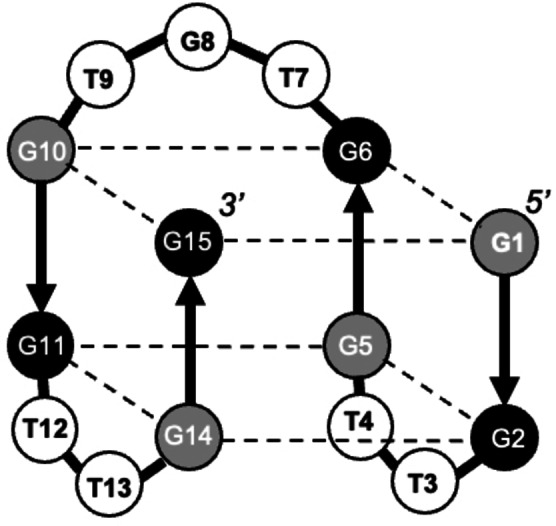
Scheme of the TBA quadruplex. The black and gray spheres correspond to *anti* and *syn* guanines, respectively, and white spheres correspond to loop nucleotides.

To begin to address this open question, in this study, we determined the stability factors of another 15‐nt quadruplex, the thrombin‐binding aptamer (TBA), GGTTGGTGTGGTTGG. TBA folds into a chair‐type antiparallel quadruplex containing two G‐tetrads connected through three short, lateral loops: one central TGT‐loop spanning the wide groove and two terminal TT‐loops spanning the narrow grooves (Figure 1). Although TBA is comprised of only two G‐tetrads, it is structurally more diverse than the G3T quadruplex and contains both *syn* (positions 1, 5, 10 and 14) and *anti* (positions 2, 6, 11 and 15) G orientations. In addition, loop nt interactions with the G‐tetrads significantly contribute to the overall stability: (i) G8 of the TGT loop stacks with the top G‐tetrad, and (ii) T4 and T13 of the terminal loops are involved in a T ⋅ T base pair.^[12]^ While the latter is true for K^+^‐TBA,^[12]^ Sr^2+^‐TBA is unable to support this base pairing interaction.^[13]^ While many aspects of DNA and RNA quadruplexes have been investigated in detail, DNA/RNA chimeric quadruplexes have been less well studied. There are few publications comparing DNA and RNA versions of TBA^[14]^ and a systematic study of single nucleotide DNA→RNA substitutions has not been reported. Here, we perform a systematic thermodynamic analysis of specific ribonucleotide substitutions in the TBA quadruplex, examining 25 constructs in total. The study revealed specific positions dictate TBA topology and stability and allowed for the design of antiparallel quadruplexes demonstrating significantly improved thermodynamic stability.

## Results and Discussion

### DNA‐TBA versus RNA‐TBA

**Figure 2:**
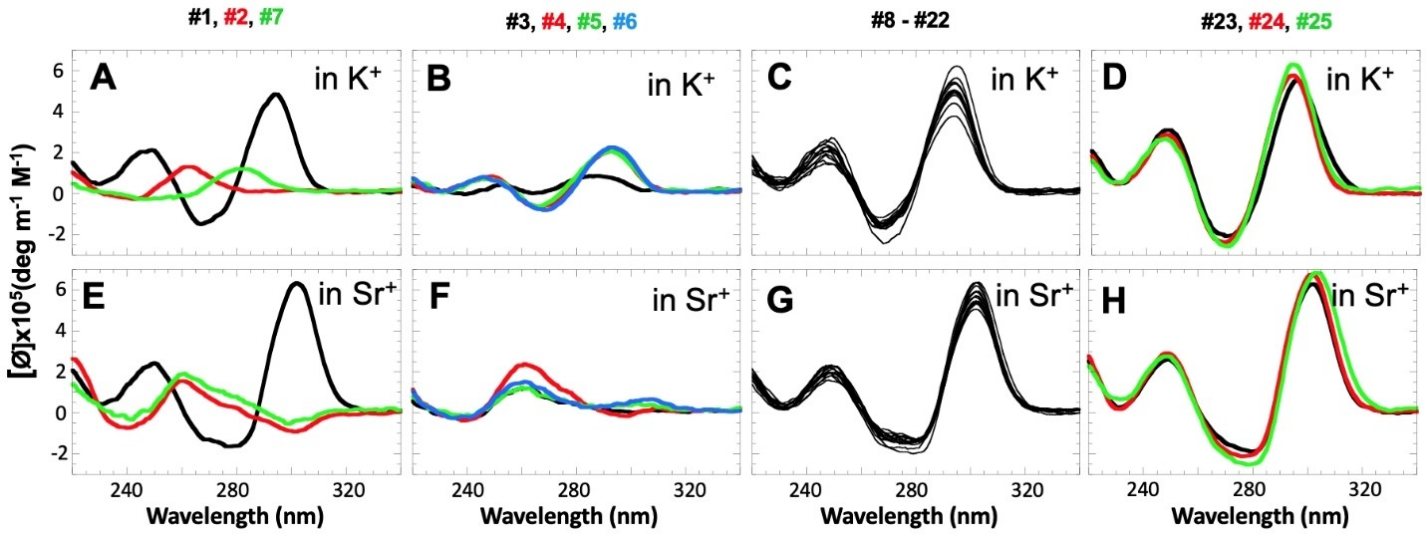
CD profiles of WT TBA and variants measured in 50 mm KCl (upper row) and 10  mm SrCl_2_ (bottom row) at 25 °C. Construct numbers correspond to the sequences shown in Table 1.

**Figure 3:**
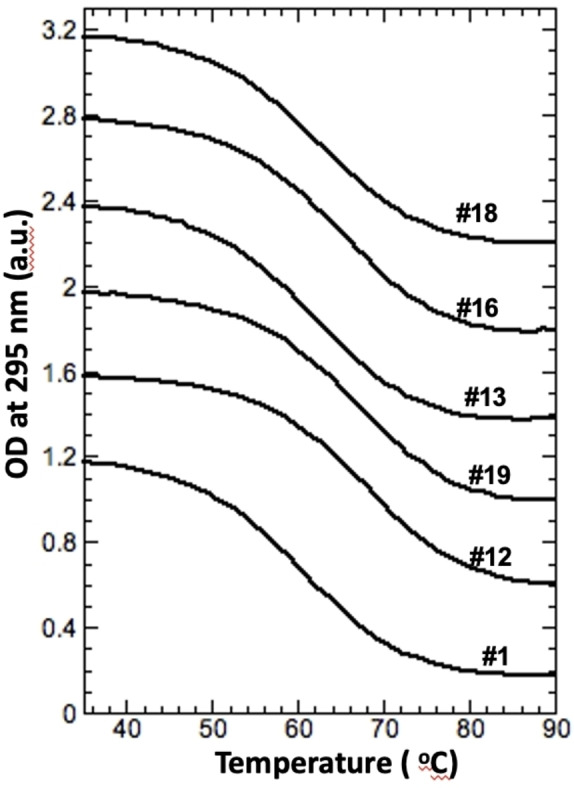
Typical UV melting curves of WT TBA and variants in 10 mm SrCl_2_. Construct numbers correspond to the sequences shown in Table 1. Curves are offset for clarity.

The CD spectra of DNA‐TBA shown in Figure 2A and E (construct #1) are characteristic of an antiparallel quadruplex with positive peaks at ≈250 nm and ≈300 nm and a negative peak at ≈270 nm. A typical UV melting profile is shown in Figure 3, revealing a sigmoidal transition with *T*_m_ of 61 °C in 10 mm Sr^2+^ (Table 1). The van't Hoff analysis revealed similar values for DNA‐TBA and all chimeric constructs (Δ*H*_vH_=39±6 kcal mol^−1^ in K^+^ and Δ*H*_vH_=35±7 kcal mol^−1^ in Sr^2+^). The *T*_m_ value of DNA‐TBA is independent of strand concentration, suggesting a monomolecular transition (Figure S2, Supporting Information).^[15]^

**Table 1:** Melting temperatures of TBA and variants.^[a]^

Oligonucleotide	Number	Designation	*T*_m_ in 50 mm K^+^	*T*_m_ in 10 mm Sr^2+^
GGTTGGTGTGGTTGG	#1	TBA	50.5	61.0
**gguuggugugguugg**	#2	RNA‐TBA	nd^[b]^	nd
**g**GTTGGTGTGGTTGG	#3	G1→g	nd	nd
GGTT**g**GTGTGGTTGG	#4	G5→g	nd	nd
GGTTGGTGT**g**GTTGG	#5	G10→g	nd	nd
GGTTGGTGTGGTT**g**G	#6	G14→g	nd	nd
**g**GTT**g**GTGT**g**GTT**g**G	#7	G1,5,10,14→g	nd	nd
G**g**TTGGTGTGGTTGG	#8	G2→g	52.0	63.5
GGTTG**g**TGTGGTTGG	#9	G6→g	51.5	64.0
GGTTGGTGTG**g**TTGG	#10	G11→g	52.0	63.0
GGTTGGTGTGGTTG**g**	#11	G15→g	52.5	63.5
**g**TTG**g**TGTG**g**TTG**g**	#12	G2,6,11,15→g	56.5	72.0
GG**u**TGGTGTGGTTGG	#13	T3→u	50.5	61.0
GGT**u**GGTGTGGTTGG	#14	T4→u	39.0	51.5
GG**uu**GGTGTGGTTGG	#15	T3,4→u	40.0	52.5
GGTTGG**u**GTGGTTGG	#16	T7→u	53.5	68.0
GGTTGGT**g**TGGTTGG	#17	G8→g	50.5	61.0
GGTTGGTG**u**GGTTGG	#18	T9→u	50.5	61.5
GGTTGG**ugu**GGTTGG	#19	TGT→ugu	53.5	69.0
GGTTGGTGTGG**u**TGG	#20	T12→u	50.5	60.5
GGTTGGTGTGGT**u**GG	#21	T13→u	37.0	46.0
GGT**U**GGTGTGGTTGG	#22	T4→dU	50.0	57.0
GGT**A**GGTGTGGTTGG	#23	T4→A	51.0	71.5
GGTTGGTGTGGT**A**GG	#24	T13→A	50.0	70.0
G**g**T**A**G**gu**GTG**g**TTG**g**	#25	Stable TBA	63.0	85.0

[a] Melting temperatures, *T*_m_ (°C) were derived from the shapes of UV melting curves measured at a concentration of ≈4 μm per strand in 10 mm Tris‐HCl, pH 8.7. Values represent the average of at least three determinations and experimental errors are estimated ±0.5 °C; [b] nd=not determined due to the noncooperative nature of the curves. Upper case letters indicate DNA and lower case letters indicate RNA. Sequence changes relative to WT TBA are indicated in bold.

In contrast, the RNA analog of TBA, RNA‐TBA, is incapable of forming an intramolecular antiparallel quadruplex in the presence of either K^+^ or Sr^2+^, as evidenced by the CD profile, which demonstrated a positive peak around 265 nm, characteristic of parallel quadruplexes (construct #2, Figure 2A, E). UV melting experiments revealed an uncooperative transition consistent with unstable multimolecular structures (**Figure S3A**). This is in accord with a previous study of RNA‐TBA^[14b]^ and numerous other studies of RNA quadruplexes.[^14a^, ^16^] These studies demonstrate that RNA quadruplexes are limited to the parallel topology due to the inability of ribonucleotides to adopt the *syn* conformation required for the antiparallel folds.

### G→g Substitutions at G‐Tetrads

**Gs with** ***syn*** **Glycosidic Bonds**. Construct #7 contains simultaneous ribonucleotide substitutions in all four Gs with *syn* glycosidic bonds (Table 1, positions 1, 5, 10 and 14). This construct was incapable of maintaining intramolecular antiparallel topology. This was true in the presence of both cations tested with some minor differences. In K^+^ solution, the quadruplex was completely destabilized above 20 °C (Figure S3B) and the CD spectrum (recorded at 25 °C) demonstrated a weak signal at 280 nm consistent with an unfolded structure (Figure 2A). In Sr^2+^ solution, the structure was slightly more stable (Figure S3B) and the CD spectrum was almost superimposable with the spectrum for RNA‐TBA, suggesting the formation of an intermolecular parallel quadruplex (Figure 2E).

The constructs with single G→g substitutions at Gs in the *syn* conformation (Table 1, #3–6) demonstrate mainly parallel topologies in the presence of Sr^2+^ ions (Figure 2F). In the presence of K^+^ ions, CD profiles are consistent with the antiparallel topology but with very low intensities (Figure 2B). Interestingly, construct #3, with a G1→g substitution, shows the smallest CD intensity, and its positive peak is shifted towards 280 nm (Figure 2B, black) resembling the construct with four G→g substitutions (#7 in Figure 2A). Thus, the destabilization effect of G1→g is comparable to four simultaneous substitutions, which we attribute to its terminal position (see Table 1). All five DNA/RNA chimeras (#3–7) demonstrated more than a 25 °C decrease in thermal stability and *T*_m_′s could not be determined due to the noncooperative nature of the transitions.

**Gs with** ***anti*** **Glycosidic Bonds**. Simultaneous substitution in all four Gs with *anti* glycosidic bonds in the presence of K^+^ resulted in 6 °C stabilization (Table 1, #12) without altering the chair‐type conformation (Figure 2C). This is in good agreement with previous studies under similar conditions.^[14b]^ Single G→g substitutions in positions 2, 6, 11 and 15 (Table 1, #8–11) did not produce any significant changes to the CD profiles (Figure 2C) and revealed moderate stabilization effects (≈1.5 °C per substitution) (Table 1). In addition, the stabilization effects produced by G→g substitutions at Gs with *anti* glycosidic bonds are additive (Table 1, constructs #8–12). The same substitutions in the presence of Sr^2+^ ions demonstrated significantly stronger stabilization effects (Table 1 and Figure 3) without affecting CD profiles (Figure 2G). Each single G→g substitution resulted in ≈2.5 °C stabilization (Table 1, #8‐11) and simultaneous substitutions in all *anti* positions resulted in 11 °C stabilization (Table 1, #12). UV melting experiments of constructs #8–12 demonstrated sigmoidal transitions characteristic of two‐state transitions (Figure 3). The *T*_m_ values of these constructs are independent of strand concentration (Figure S2), which indicates formation of monomolecular structures.

### Substitutions in the Central TGT Loop

In the presence of both cations, DNA→RNA substitutions within the TGT‐loop did not affect the antiparallel topology of TBA (Figure 2, #16–19). Significant changes in thermal stability were observed only in the case of T7→u; 3 °C and 7 °C for K^+^ and Sr^2+^, respectively (Table 1 and Figure 3, #16). The substitutions at positions 8 and 9 did not reveal any measurable effects in the presence of either cation (Table 1, #17,18). Both X‐ray and NMR studies revealed that while T7 is fully exposed to solution, G8 and T9 are stacked on the adjacent G‐tetrad.^[17]^ Thus, DNA→RNA substitutions at nt positions involved in stacking interactions with the adjacent G‐tetrad produced no measurable stability effects. However, the substitution at fully solvated position 7 demonstrated a significant increase in stability. Interestingly, all T‐loops in the G3T quadruplex are also fully exposed to solution without any interaction with G‐tetrads, and demonstrated a ≈1.5 °C increase in stability upon T→u substitutions.^[11]^

### Substitutions in the Terminal TT Loops

**Figure 4:**
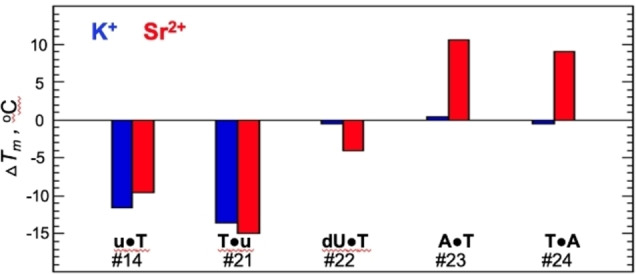
Bar graph summarizing stability effects of constructs with substitutions in positions 4 or 13 relative to WT TBA in K^+^ (blue) and Sr^2+^ (red). Construct numbers correspond to the sequences shown in Table 1.

T→u substitutions at positions 3 and 12 (constructs #13 and #20, respectively) did not reveal any measurable effects in either K^+^ or Sr^2+^ solutions (Table 1 and Fig 2 C, G). However, substitutions at positions 4 and 13 (constructs #14 and #21) revealed strong destabilization. T4→u substitution resulted in an 11.5 °C and 9.5 °C decrease in *T*_m_ for K^+^ and Sr^2+^, respectively. Even greater destabilization effects, 13.5 °C and 15 °C, were observed with the T13→u substitution (Table 1 and Figure 4).

***K*** ^***+***^ ***‐TBA***. A previous NMR study of K^+^‐TBA revealed that T3 and T12 are not involved in any interaction with the quadruplex and are completely exposed to solution.^[12]^ In contrast, T4 and T13 are stacked on the adjacent G‐tetrad forming a parallel T ⋅ T mismatch with two hydrogen bonds.^[12]^ Since T→u substitution is not expected to disturb the base pairing and all loop nt of wild type (WT) TBA are in the *anti* conformation,^[12]^ the observed destabilization effects may be attributed to the sugar pucker of the ribose. The ribose favors a 3′‐*endo* sugar conformation, which results in a 5.9 Å inter‐phosphate distance, compared to a 7.0 Å distance in the case of deoxyribose, which favors a 2′‐*endo* conformation. Thus, the destabilization effect of T→u substitutions at positions 4 and 13 may be attributed to the limited ability of Tu‐loops, versus TT‐loops, to span the quadruplex grooves. This proposal is supported by T4→dU substitution, which restores the stability of the quadruplex to that of the WT K^+^‐TBA (Figure 4 and Table 1, #22). In addition, T4→C or T13→C substitutions in K^+^‐TBA resulted in only ≈6 °C destabilization.^[18]^ The moderate destabilization effect of T→C versus T→u substitution is likely due to the disruption of T ⋅ T hydrogen bonds and possible destacking from the adjacent G‐tetrad.

To further study the base pairing capability of other nt at positions 4 and 13, we tested T→G and T→A substitutions. Quadruplexes with T→G substitutions displayed some structural polymorphism, which complicated unambiguous interpretation of the data, and these variants were not characterized further. In contrast, T→A substitutions at these positions did not affect the chair‐type topology of K^+^‐TBA as the CD spectrum demonstrated an identical profile with a 10–20 % increase in amplitude relative to WT TBA (constructs #23 and #24, Figure 2D, H). These substitutions also did not affect the thermal stability of TBA (Table 1 and Figure 4). This might indicate that parallel T ⋅ T mismatches and parallel A ⋅ T base pairs (likely reverse Watson‐Crick) contribute similarly to the overall stability of the quadruplex.

***Sr*** ^***2+***^ ***‐TBA***. NMR studies revealed that while K^+^‐TBA and Sr^2+^‐TBA quadruplexes adopt the same chair‐type topology, there are significant structural differences. For instance, the inter‐tetrad distance of the Sr^2+^‐TBA quadruplex is 0.7 Å larger than the K^+^‐TBA quadruplex.^[13]^ In addition, in Sr^2+^‐TBA, T4 and T13 do not form a base pair and are not co‐planar with the adjacent G‐tetrad as they are in K^+^‐TBA.^[13]^ Thus, the destabilization effect of T→u substitutions in Sr^2+^‐TBA (9.5 °C for T4→u and 15 °C for T13→u) (Figure 4, #14 and #21) is likely attributed to the constraint on Tu‐loops spanning the grooves. In addition, the T4→dU substitution almost completely restores the thermodynamic stability to that of WT Sr^2+^‐TBA. While the T4→A and T13→A substitutions are not accompanied by any measurable effects on the *T*_m_ of K^+^‐TBA, they significantly stabilize Sr^2+^‐TBA (Table 1, #23 and #24). This clearly indicates that the geometry of the Sr^2+^‐TBA quadruplex allows for parallel A ⋅ T base pair formation but not the T ⋅ T mismatch in positions 4 and 13. In contrast, K^+^‐TBA accepts both pairs equally. Thus, while substitutions at positions 4 and 13 did not result in increased thermal stability of K^+^‐TBA, they did produce an ≈10 °C increase in stability of Sr^2+^‐TBA (Figure 4).

### Mixed RNA/DNA Substitutions

Based on the results described above, and assuming stabilization effects are cumulative, we hypothesized that a construct with five DNA→RNA substitutions (G2→g, G6→g, G12→g, G15→g and T7→u) and one DNA→DNA (T4→A) substitution may result in a TBA quadruplex with significantly (≈10 °C for K^+^ and ≈28 °C for Sr^2+^) improved thermal stability. The CD spectra of this construct (Table 1, #25) correspond to a chair‐type topology (Figure 2D, H). Experimentally measured stabilization effects were close to the predicted values, yielding 12.5 °C and 24 °C increased stability relative to WT TBA for the K^+^ and Sr^2+^ quadruplexes, respectively (Table 1).

## Conclusion

**Figure 5:**
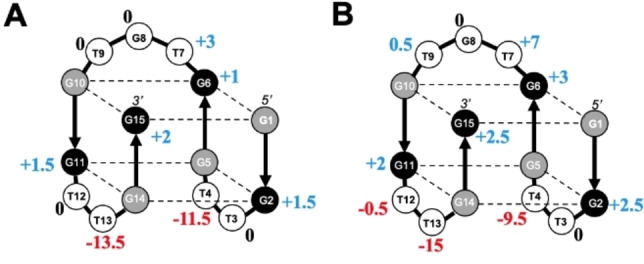
Summary of stability effects (Δ*T*_m_) of single‐nucleotide dG→g and dT→u substitutions in the presence of K^+^ (A) and Sr^2+^ (B). Increases in stability are shown in blue and decreases are in red. No change is indicated in black. The black and gray spheres correspond to *anti* and *syn* guanines, respectively, and white spheres correspond to loop nucleotides.

Figure 5 summarizes the results of this study by indicating Δ*T*_m_ values of single‐nt DNA→RNA substitutions on the chair‐type topology of TBA. Our study revealed that all DNA/RNA chimeras with G→g substitutions in G residues requiring *syn* conformation (grey spheres at positions 1, 5, 10 and 14) demonstrated strong destabilization in the presence of either K^+^ or Sr^2+^, prohibiting thermodynamic analysis of the constructs. The G→g substitutions at Gs with *anti* conformation (black spheres at positions 2, 6, 11 and 15) increased the stability of both K^+^‐TBA and Sr^2+^‐TBA without affecting the monomolecular chair‐type topology. Stabilization effects at all four positions were found to be similar (≈1.5 °C and ≈2.5 °C for K^+^ and Sr^2+^, respectively).

None of the DNA→RNA substitutions in loop positions affected the monomolecular chair‐type topology of the quadruplexes. However, Δ*T*_m_ values varied from −13.5 °C to +3 °C for K^+^‐TBA and −15 °C to +7 °C for Sr^2+^‐TBA. Interestingly, two substitutions (T4→u and T13→u) in the terminal loops resulted in strong destabilization, which is attributed to the differential ribose sugar puckering. This difference likely limits the capability of Tu‐loops to span across the quadruplex grooves. Another source of destabilization comes from breaking the T4 ⋅ T13 base pair formed in K^+^‐TBA, but not in Sr^2+^‐TBA.

These data allowed us to design the chimeric construct, G ***g*** T ***A*** G ***gu*** GTG ***g*** TTG ***g***, containing five ribonucleotides resulting in a 12.5 °C (in K^+^) and 24 °C (in Sr^2+^) increase in stability relative to the WT TBA. The knowledge gained from this work can be used to design quadruplexes with improved thermodynamic properties for a variety of applications including both static and dynamic nanostructures.^[19]^

## Experimental Section

All DNA oligonucleotides were obtained from Integrated DNA Technologies. Concentrations were determined by measuring UV absorption at 260 nm, as described previously.^[20]^ All measurements were performed in 10 mm Tris‐HCl, pH 8.7, with 50 mm KCl or 10 mm SrCl_2_. UV unfolding/folding experiments were measured at 295 nm using a Varian UV‐visible spectrophotometer (Cary 100 Bio). Circular dichroism (CD) spectra were obtained using a Jasco‐815 spectropolarimeter. Both devices were equipped with thermoelectrically controlled cuvette holders. In a typical experiment, oligonucleotide stock solutions were mixed into their desired buffers in optical cuvettes. The solutions were then incubated at 95 °C for several min and placed at room temperature for 10–15 min before ramping to the desired starting temperature of 20 °C. Melting experiments performed at a heating rate of 1 °C min^−1^ resulted in superimposable heating and cooling curves, confirming equilibrium transitions (Figure S1). CD and UV experiments were conducted at 4 μm concentration of the TBA construct. Temperature‐dependent UV absorbance curves allowed the *T*_m_ to be deduced from the temperature corresponding to the midpoint of the unfolding process. Van't Hoff enthalpies, Δ*H*_vH_, were calculated using the following equation: Δ*H*_vH_=4 *R* *T*_m_^2^ δα/δ*T* where *R* represents the gas constant and δα/δ*T* is the slope of the normalized optical absorbance versus temperature curve at the *T*_m_.^[21]^ The *T*_m_ values and CD profiles represent an average of at least three measurements.

## Conflict of interest

The authors declare no conflict of interest.

1

## Supporting information

As a service to our authors and readers, this journal provides supporting information supplied by the authors. Such materials are peer reviewed and may be re‐organized for online delivery, but are not copy‐edited or typeset. Technical support issues arising from supporting information (other than missing files) should be addressed to the authors.

Supporting Information

## Data Availability

The data that support the findings of this study are available from the corresponding author upon reasonable request.
