# Supplementary material for: A Chimeric DNA/RNA Antiparallel Quadruplex with Improved Stability
Source: ChemistryOpen. 2022 Feb 1;11(2):e202100276. doi: 10.1002/open.202100276 (PMC8805387; doi:10.1002/open.202100276)
Supplement: Supplementary file 1 — Supporting Information [file OPEN-11-e202100276-s001.pdf]

ChemistryOpen

Supporting Information

A Chimeric DNA/RNA Antiparallel Quadruplex with Improved Stability

Elaina P. Boyle, Levan Lomidze, Karin Musier-Forsyth, and Besik Kankia*

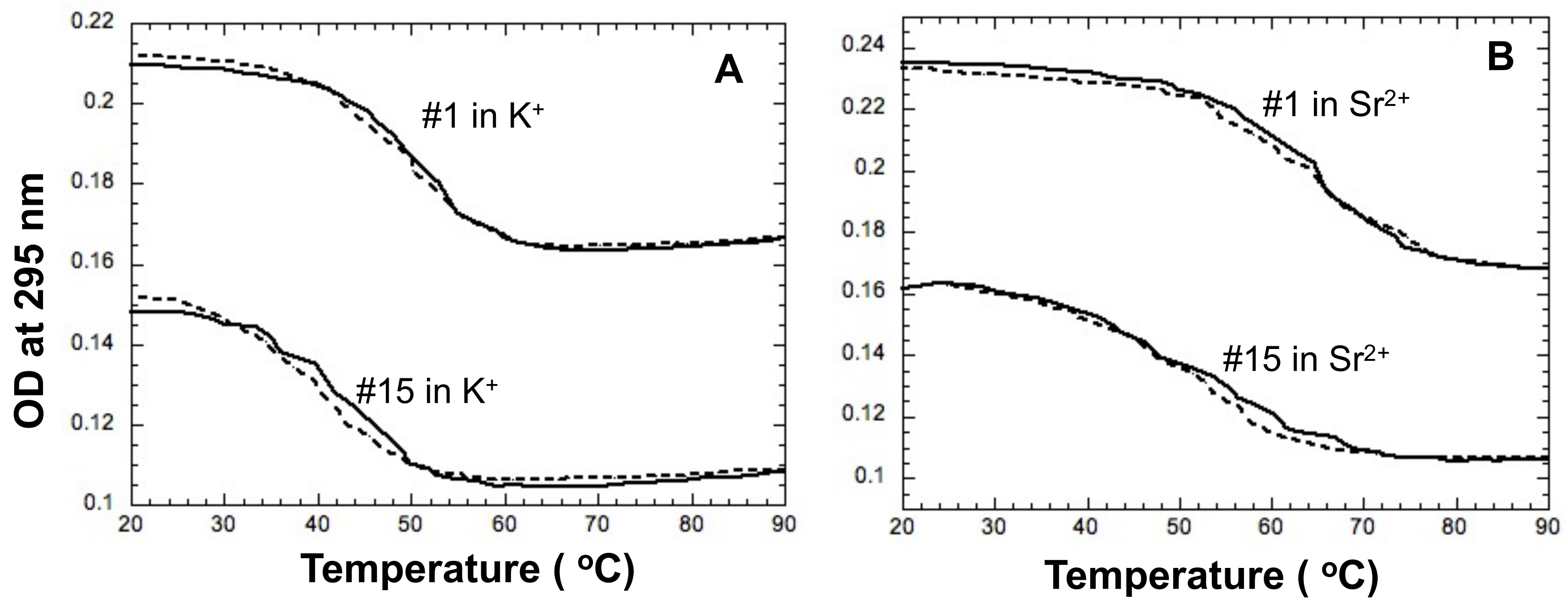

#1 GGTTGGTGTGGTTGG
#15 GG**uu**GGTGTGGTTGG

Figure S1. UV melting experiments. Representative heating (solid) and cooling (dashed) curves of DNA-TBA (construct #1) and T3,4$\rightarrow$u (construct #15). Measurements were performed in 50 mM KCl (**A**) and 10 mM $SrCl_2$ (**B**) at 4 µM strand concentration. Buffer: 10 mM Tris, pH 8.7.

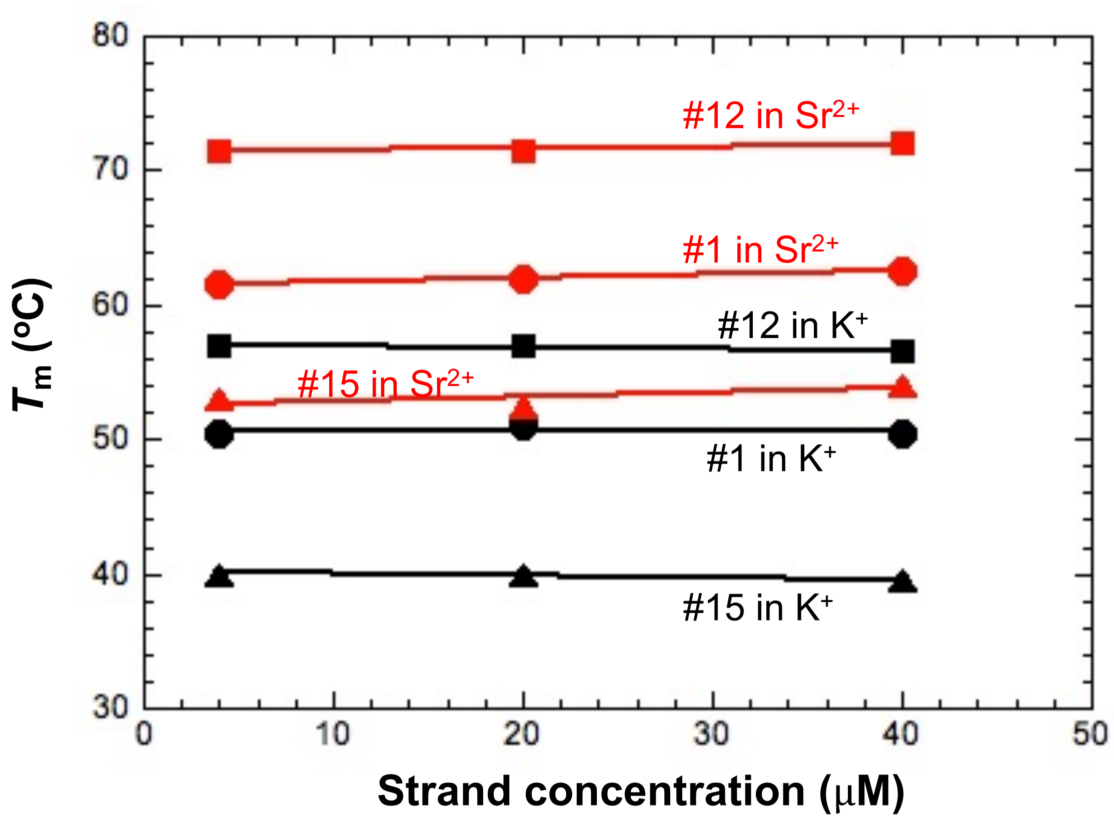

#1 GGTTGGTGTGGTTGG
#12 G**g**TTG**g**TGTG**g**TTG**g**
#15 GG**uu**GGTGTGGTTGG

Figure S2. Tm dependence on strand concentration for DNA-TBA (construct #1), G2,6,11,15→g (construct #12) and T3,4→u (construct #15) sequences. Measurements were performed in 50 mM KCl (black) and 10 mM $SrCl_2$ (red). Buffer: 10 mM Tris, pH 8.7.

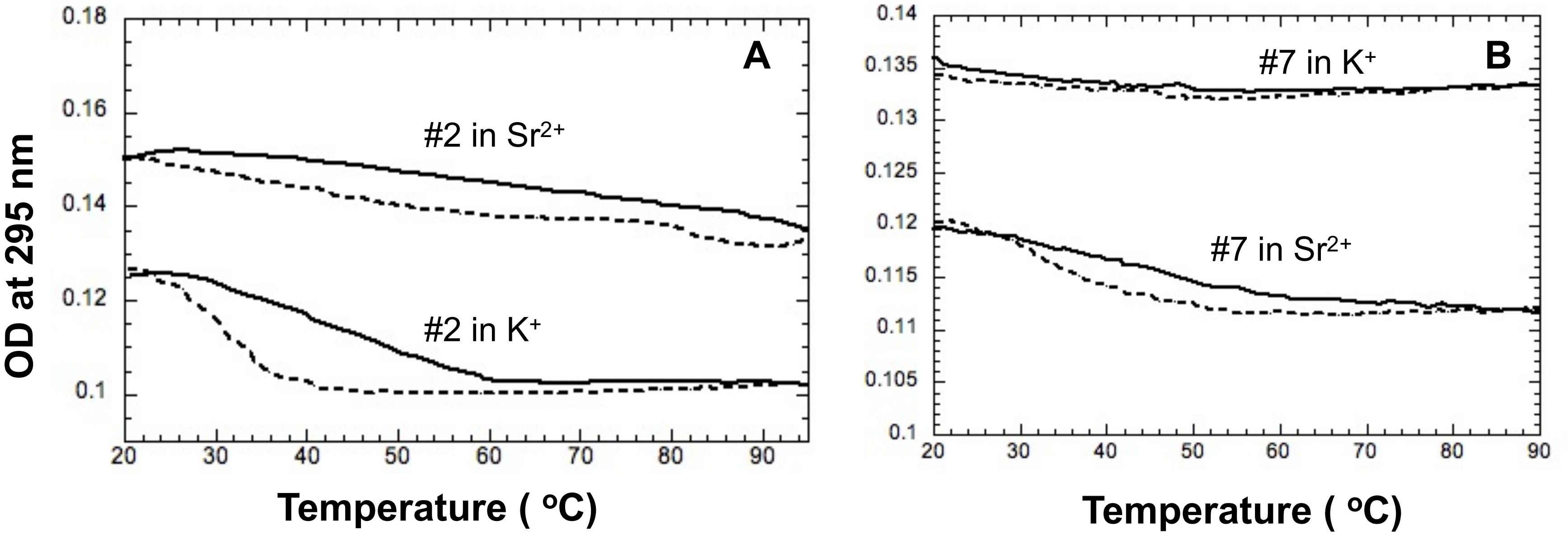

#2 **gguuggugugguugg**

#7 **g**GTT**g**GTGT**g**GTT**g**G

Figure S3. UV melting experiments. Representative heating (solid) and cooling (dashed) curves of RNA-TBA (construct #2) (**A**) and G1,5,10,14→g (construct #7) (**B**). Measurements were performed in 50 mM KCl and 10 mM $SrCl_2$ at 4 µM strand concentration. Buffer: 10 mM Tris, pH 8.7.
